# Research on the Method of Counting Wheat Ears via Video Based on Improved YOLOv7 and DeepSort

**DOI:** 10.3390/s23104880

**Published:** 2023-05-18

**Authors:** Tianle Wu, Suyang Zhong, Hao Chen, Xia Geng

**Affiliations:** College of Information Science and Engineering, Shandong Agricultural University, Tai’an 271018, China; tianlewu2002@163.com (T.W.);

**Keywords:** wheat-ear counting, improved YOLOv7, DeepSort, unmanned aerial vehicle (UAV)

## Abstract

The number of wheat ears in a field is an important parameter for accurately estimating wheat yield. In a large field, however, it is hard to conduct an automated and accurate counting of wheat ears because of their density and mutual overlay. Unlike the majority of the studies conducted on deep learning-based methods that usually count wheat ears via a collection of static images, this paper proposes a counting method based directly on a UAV video multi-objective tracking method and better counting efficiency results. Firstly, we optimized the YOLOv7 model because the basis of the multi-target tracking algorithm is target detection. Simultaneously, the omni-dimensional dynamic convolution (ODConv) design was applied to the network structure to significantly improve the feature-extraction capability of the model, strengthen the interaction between dimensions, and improve the performance of the detection model. Furthermore, the global context network (GCNet) and coordinate attention (CA) mechanisms were adopted in the backbone network to implement the effective utilization of wheat features. Secondly, this study improved the DeepSort multi-objective tracking algorithm by replacing the DeepSort feature extractor with a modified ResNet network structure to achieve a better extraction of wheat-ear-feature information, and the constructed dataset was then trained for the re-identification of wheat ears. Finally, the improved DeepSort algorithm was used to calculate the number of different IDs that appear in the video, and an improved method based on YOLOv7 and DeepSort algorithms was then created to calculate the number of wheat ears in large fields. The results show that the mean average precision (mAP) of the improved YOLOv7 detection model is 2.5% higher than that of the original YOLOv7 model, reaching 96.2%. The multiple-object tracking accuracy (MOTA) of the improved YOLOv7–DeepSort model reached 75.4%. By verifying the number of wheat ears captured by the UAV method, it can be determined that the average value of an L1 loss is 4.2 and the accuracy rate is between 95 and 98%; thus, detection and tracking methods can be effectively performed, and the efficient counting of wheat ears can be achieved according to the ID value in the video.

## 1. Introduction

The number of wheat ears in a field is a key indicator for evaluating the overall wheat yield, which is related to national food security and economic efficiency factors. Hence, the timely and accurate counting of wheat ears is of great practical importance for yield predictions. The traditional, manual, statistical sampling method used in the research is not only time-consuming and laborious but also produces a low accuracy rate. In recent years, with the rapid development of artificial intelligence, intelligent agricultural production management has become a new goal and general direction for agricultural development.

Although traditional machine learning techniques used for crop yield estimations address the shortcomings of manual methods, to some extent, they still suffer from numerous problems, such as exhibiting unclear image demarcations as a result of sliding window redundancies [1], complex target feature designs, poor portability, and cumbersome manual designs [2]. Therefore, an increased number of deep learning-based target-detection algorithms are used in the field to count wheat ears, which mainly contain two-stage algorithms, represented by the R-CNN (regions with CNN features) series [3], and single-stage algorithms, represented by the YOLO (you only look once) series [4].

In terms of two-stage-based target-detection methods, Hasan et al. [5] and Li et al. [6] adopted the use of R-CNN networks in their research to implement wheat-recognition training. Although the average recognition accuracy value was as high as 93.4% for wheat-ear counting, it was unavailable for real-time detection owing to the slow pace of detection. Zhao et al. [7] proposed a deep neural network-based wheat-ear-detection method with an AP (average precision) value of 94.5% for real-time detection results. David et al. [8] established a global wheat head detection (GWHD) dataset in 2021, which has the advantage of producing less noise and assessing more samples compared to GWHD_2020. From the perspective of single-stage wheat-ear-based target-detection methods, single-stage target-detection models have significantly progressed in the field of wheat-ear image-detection counting methods (Madec et al. [9], 2019; He et al. [10], 2020) and are also the best wheat-detection devices, at present, in terms of their detection accuracy and speed (Khoroshevsky et al. [11], 2021; Lu et al. [12], 2021). To solve the problem of the low accuracy factor of low target detection in images, Yang et al. [13] proposed an improved YOLOv4 model with an attention mechanism that can enhance the feature-extraction capability of the network by adding an acceptance domain module. Zhao et al. [7] proposed a deep learning method (OSWSDet) for the orientation and detection of small-target wheat ears with an AP value of 90.5%. Zhao et al. [14] proposed an improved YOLOv5-based method to accurately detect the number of small wheat ears in unmanned aerial vehicle (UAV) images, and the average accuracy (AP) of wheat-ear detection in the UAV images was 94.1%, which was 10.8% higher than the standard YOLOv5 method, largely solving the problem of incorrect and missed wheat-ear-detection outcomes.

The assays used at present for wheat ears are all based on earlier YOLO versions mentioned above, including YOLO9000 (Redmon and Farhadi [15], 2017), YOLOv3 (Redmon and Farhadi [16], 2018), YOLOv4 (Bochkovskiy et al. [17], 2020), and YOLOv5 (Ultralytics [18], 2021). YOLOv7, proposed by Wang et al. [19], presents greater detection accuracy and rate values than the previous YOLO series.

The above-mentioned counting studies are all conducted by using static wheat-ear images, while in a large field environment, the problems of mutual occlusion and overlapping of wheat-ear images exist. In addition, the visual angle of wheat-ear images is singular and limited, which leads to some wheat ears not being visible in the images, thus causing a high counting error. Even if a video of wheat ears is taken using UAVs, the video data must be converted into image data through certain processes, such as key frame selection and image stitching, resulting in poor accuracy produced by image mosaicking, which directly affects the accuracy of subsequent wheat-ear counting results. Moreover, the video data first need to be converted into image data; the efficiency of wheat-ear counting is then also reduced. Therefore, this paper studies the method of wheat-ear counting directly based on video methods, which can obtain wheat-ear information from multiple angles and better present the state of wheat ears in a field. Video-based wheat counting methods can produce higher robustness and counting efficiency outcomes.

At present, video-based fruit-counting methods are combined with multi-target tracking algorithms based on target-detection methods. Osman et al. [20] performed the dynamic tracking and counting of apples with an accuracy of 91% and performed the good tracking of targets with restricted fields of view under occlusion and static conditions. Ge et al. [21] detected and tracked tomatoes during different periods, including flowering, green, and red tomatoes with accuracy values of 93.1%, 96.4%, and 97.9%, respectively. Zheng et al. [22] video filmed citrus fruits by UAV, and counted the citrus appearing in the video with an F1 score of 89.07% by combining the YOLO and DeepSort models. At present, the studies performed on fruit-counting methods are mostly aimed at larger fruits, such as apples, tomatoes, and oranges; however, fewer studies on the video-based counting of smaller and densely distributed wheat ears exist in the literature.

In summary, the target-detection method, on the one hand, is at the basis of the video-based multi-target tracking algorithm; improvements have been made in the field, according to YOLOv7. Previous studies have shown that it can effectively enhance the interaction between different dimensions, as well as improve the model’s accuracy for detecting appropriate targets, by improving the feature-extraction capability of the model. Xu et al. [23] proposed the SR-YOLOv5 model on the basis of YOLOv5 to improve the model’s feature-extraction capability in relation to human faces, resulting in a face recognition accuracy of 96.3%. Quoc et al. [24] improved the model’s feature-extraction capability when detecting human ears, resulting in an accuracy rate of 98.7%. Hence, this study aims to improve the detection accuracy of YOLOv7 for use in wheat-ear detection studies. Firstly, in the network structure, ODConv (full-dimensional dynamic convolution) [25] is introduced to significantly improve the feature-extraction capability of the model, enhance the inter-dimensional interaction, and improve the performance of the detection model. Secondly, GCNet (global context network) [26] is added to the backbone network to perform the efficient modeling of global information. Finally, the CA (coordinate attention) [27] mechanism is adopted to enhance the direction-related location information and achieve effective feature-extraction results. On the other hand, the DeepSort [28] multi-target tracking algorithm is improved in this study, The DeepSort model’s feature extractor was originally designed for pedestrians and is not applicable to wheat ears, while ResNet [29] has the characteristics of being lightweight and producing accurate results. This study replaces the DeepSort model’s feature extractor with the improved ResNet network. Accordingly, an improved YOLOv7 and DeepSort-based video-counting method for use in large fields of wheat is achieved by counting the number of different IDs in the video.

## 2. Materials and Methods

**Figure 1:**
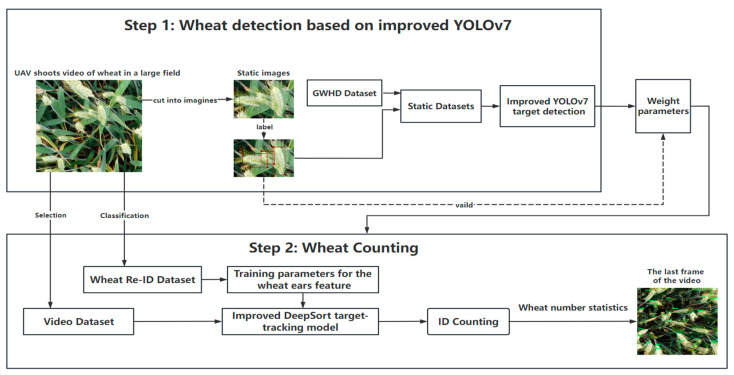
Flowchart of wheat-ear counting method.

The overall process of video-based wheat-ear counting presented in this paper is shown in Figure 1. The video of wheat ears in a large field taken by an unmanned aerial vehicle was input, and the improved YOLOv7 model was used to detect and identify wheat ears frame by frame. The detection result was sent to the tracking module, which is responsible for building the link between frames to track one target. When the entire video is processed, a number of different ID values are obtained, which represent the required number of wheat ears.

### 2.1. Dataset Creation

The data presented in this paper were derived from two areas: the static image data of GWHD provided by the International Conference on Computer Vision, and the video data of field wheat ears collected by UAV in the experimental plots. The GWHD is a large, diverse, and well-labeled wheat-ear dataset. However, we observed, after careful screening, there were a few missing and incorrect labels in GWHD. As a result, this study only selected the well-labeled data obtained from GWHD to form the dataset. In addition, the data collected by UAV were highly targeted for different types of wheat ears due to their location in the real environment of a wheat field. Therefore, compared with a single-source dataset, the dataset composed of data obtained from two sources can better address the physical signs of wheat during different growing cycles and the complex environmental differences in the field, so as to improve the robustness and accuracy of the model counting the wheat ears. The dataset details are presented in Table 1.

**Construction of wheat-ear dataset based on GWHD**

GWHD contains 4700 high-resolution RGB images and 190,000 labeled wheat ears. Due to the phenomena of occlusion, complex background, and large variation in the scale of wheat ears, this paper selected 2600 images with considerable differences, eliminated similar images to ensure the diversity of the dataset, checked the labeling effect, and supplemented the labeling of missing wheat ears with inconspicuous visual features to avoid the possibility of incorrect and missing labels as much as possible.

**Construction of wheat-ear dataset based on unmanned aerial vehicle collection**

The data collection site was located at the Agronomy Experiment Station, Panhe Campus, Shandong Agricultural University, Tai’an, Shandong Province (117°9′ E, 36°9′ N), at an altitude of approximately 120 m, with a temperate monsoon climate suitable for wheat cultivation and growth.

**Figure 2:**
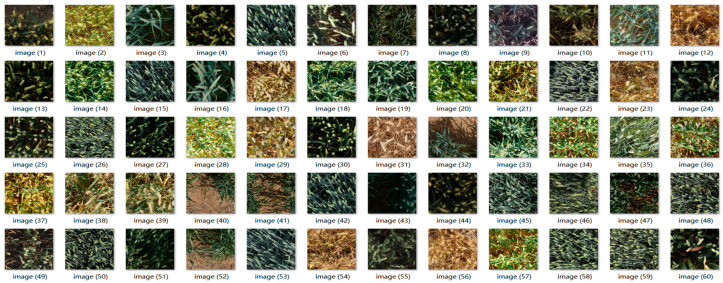
Partial images in the dataset.

Firstly, a UAV was used to shoot the video of wheat in the field. While paying attention to the resolution, it functioned at a uniform speed. Secondly, the ffmpeg tool was adopted to process the video frame by frame. Then, it was cut into images, and the photos with blurred pixels and too dense or sparsely distributed images of wheat ears were eliminated. Among them, various types of images of wheat ears in the field were produced: 1056 pictures with severe obscurations, 724 pictures of different sizes, 482 images with uneven illuminations, and 248 images of the rest of the environment. Then, the Labelimg tool was used to label the wheat ears in the images to avoid mislabeling and omissions as much as possible. Moreover, the 4200 sieved images were divided into 3350 images in the training set, 550 images in the test set, and 300 images in the validation set, some of which are presented in Figure 2.

There were two reasons for obtaining the video data: one was to cut part of the video data into frames so that the clear images could be selected to expand the dataset for wheat-ear-detection purposes; the other was to select the videos with clear images shot at a uniform speed to verify the L1 loss and counting accuracy of the algorithm for counting the number of wheat ears in a large field and the real number of wheat ears.

### 2.2. The Improvement of the YOLOv7 Model

YOLOv7, the latest generation single-shot detector (SSD) in the YOLO series, has an internal core architecture similar to YOLOv5, which mainly consists of four parts: input, backbone network, neck module, and head module. During the detection process, the image is first pre-processed by operations, such as input and data enhancements, and then the processed image is sent to the backbone network, which performs feature extraction on the image and fuses the extracted feature information through the neck module to obtain large-, medium-, or small-sized images. Eventually, the fused feature information is sent to the head of the network for detection, and the detection result is output following the completion of the head detection process.

The backbone network of YOLOv7 mainly consists of convolution, E-ELAN, SPPCSPC, and MPConv modules. The E-ELAN module adopts the ideas of Expand, Shuffle, Merge, and Cardinality to enhance the learning ability of the network without destroying the gradient path. The MPConv module adopts the MaxPool operation to expand the feature layer and fuse it with the feature information following regular convolution processing as a way to improve the generalization performance of the network-recognition step. The SPPCSPC module introduces parallel multiple MaxPool operations, thus avoiding the distortion of the image during the processing stage. Similar to YOLOv5, the neck of YOLOv7 adopts a PAFPN [30] structure. Additionally, large, medium, and small sizes of IDetect detection heads are selected as the detection head, which correspond to the three-feature information sizes after the neck module processing step is completed.

This paper mainly improved the backbone and neck modules of the YOLOv7 target-detection model. By adding ODConv to the backbone network structure, at the cost of increasing a certain number of parameters, the network’s ability to extract features was greatly improved, and the dynamic characteristics in the null domain, input channel, and output channel were expanded. When extracting the relevant features from the backbone network and fusing features obtained from the neck network, GCNet lightweight architecture is added to encode the global information for features in each location, and the absolute size and border coordinates of the regression target are then established by using a global correlation layer. The CA (coordinate attention) mechanism is added to the torso network structure to enhance the ability of the network to extract the relevant features of the wheat ears.

#### 2.2.1. GCNet

However, problems in the processing of wheat-ear images still exist, as well as the counting methods, including the differences in the target sizes of the ears, varying markers during different seasons, and severe adhesion properties.

**Figure 3:**
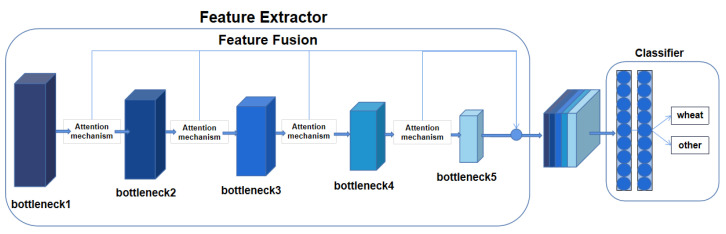
GCNet framework.

To keep a watchful eye on the system and increase its detection capability, this paper incorporated GCNet (global context network) into the YOLOv7 model, thus enhancing the extraction capability of the image features. The network structure consisted of a feature extractor and classifier, including bottleneck, attention mechanism module, fully connected layer, and SoftMax, as shown in Figure 3.

Firstly, the model was used to present the association between each element in the wheat-ear feature map, present the significance of every local feature, and reduce the influence of distracting factors. The features extracted from each bottleneck in the network are represented as *F_i_* of size N × C × W × H. The attention mechanism module is constructed using the Gram matrix [31] and the features *F_i_* are multiplied by *F_i_^T^* to obtain the local features *F^i^*_local_, as shown in Equation (1):

(1) $$F_{\mathrm{local}}^{i}=F_{i}^{T}F_{i}$$

Then, global average pooling (*GAP*) [32] is performed on feature *F_i_* in Equation (2) to obtain a global feature *F^i^*_global_ of size N × C × 1 × 1 as a way to preserve the spatial and semantic information concerning feature *F_i_*:

(2) $$F_{\mathrm{global}}^{i}=GAP(F_{i})$$

Finally, we multiplied local feature *F^i^*_local_ with global feature *F^i^*_global_ by using Equation (3) to obtain the desired overall feature *F^i^*_fusion_:

(3) $$F_{\mathrm{fusion}}^{i}=F_{\mathrm{local}}^{i}F_{\mathrm{global}}^{i}$$

Compared with the existing multi-core dynamic convolutional correspondence networks in the field, the number and quality of the features extracted were greatly increased without introducing too many additional parameters.

#### 2.2.2. ODConv

**Figure 4:**
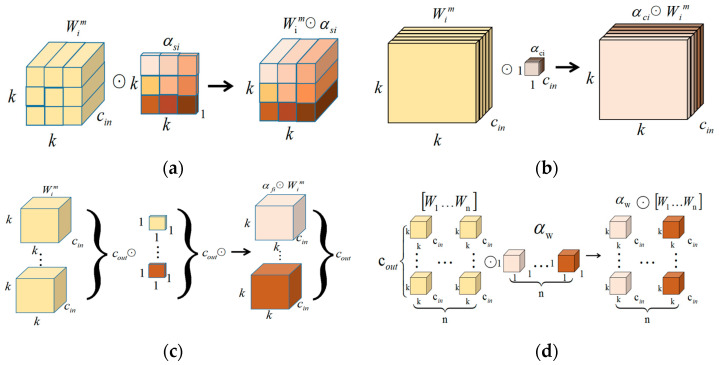
Four types of attention mechanisms in the ODConv network multiplied by the convolution kernel. (**a**) Multiplication of different positions along the spatial dimension, (**b**) multiplication operations along the input channel dimension, (**c**) filtering multiplication operations along the output channel dimension, and (**d**) kernel-wise multiplication along the kernel dimension of the convolution kernel space [25].

The ODConv (omni-dimensional dynamic convolution) network has three more dimensions than the traditional convolution network, which are the input channel, output channel, and dynamic null domain dimensions. Four multiplication operations conducted under different dimensions are presented in Figure 4. The ODConv network is a coordinate attention mechanism that can learn four dimensions of kernel space using a multidimensional attention mechanism through a parallel strategy. It can better adapt to irregularly shaped objects and backgrounds, and improve the robustness and accuracy factors of feature representation. Its core idea is to create a convolution operation that is more adaptable to irregular target shapes and backgrounds by dynamically adjusting the shape and position of the convolution kernel, assigning different attention values to the convolution filters of different channels, and thus dynamically adjusting the shape and position of the convolution kernel according to the features of the input data to substantially improve the feature-extraction capability of the convolution. More importantly, the ODConv network, with fewer convolutional kernels, can achieve a comparable, or even better, performance than CondConv [33] and DyConv [34] networks.

#### 2.2.3. CA Mechanism

**Figure 5:**
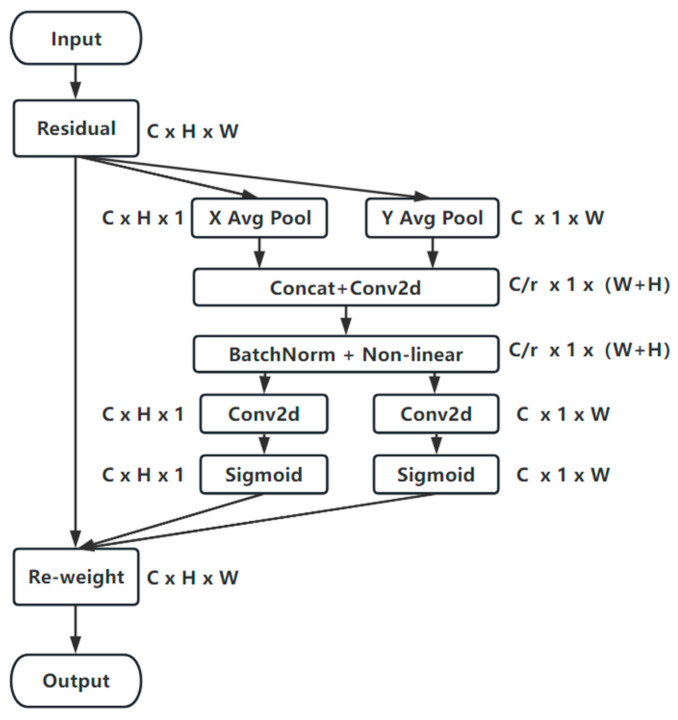
CA mechanism algorithm process.

The accuracy of the wheat-ear-detection method has an immediate impact on the wheat-tracking effect. Here, the CA mechanism was adopted to produce better effects after the consideration of the effects of varying attention mechanisms. Not only can it capture channel information, but it can also make allowances for direction-related location information and enhance the ability to learn features, thus guaranteeing the accurate location and identification of target objects. Additionally, it performs overhead computing and is flexible and lightweight. The algorithm flow is presented in Figure 5.

Firstly, the global average pooling value is decomposed. The input feature map of size C × H × W is pooled into X and Y directions, and feature maps of sizes C × H × 1 and C × H × W are generated. Then, the generated C × 1 × W feature map is transformed and obtained by performing concat, F1 (dimensionality reduction using a 1 × 1 convolution kernel), and activation operations with the C × H × 1 feature map, splitting it along the spatial dimension, and increasing the dimensionality using the convolution kernel, and finally combining it with the Sigmoid activation function to obtain the desired attention vector.

#### 2.2.4. Network Architecture Diagram of the Improved YOLOv7 Model

**Figure 6:**
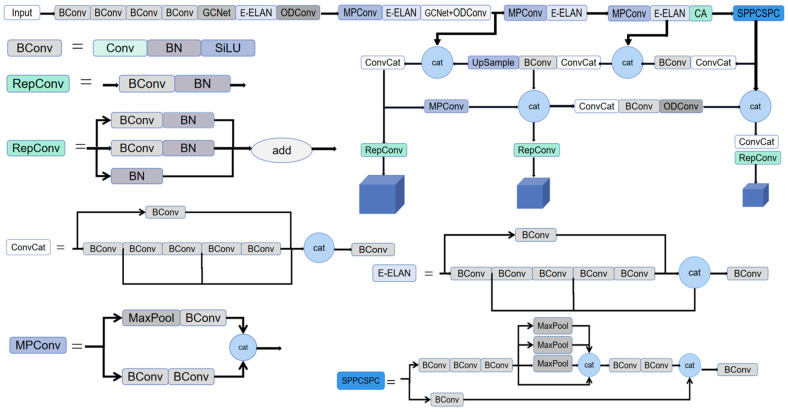
The overall network structure of the improved YOLOv7 model.

The overall improved YOLOv7 model framework is presented in Figure 6. The images in the dataset produced in this study were first fed into the improved YOLOv7 model, frame by frame, for training; then, the training weights of the improved network were obtained after training the specified number of rounds using the officially provided pre-training weights; and, finally, the improved network was evaluated using the test set images.

The improved YOLOv7 model connects each pixel in an image with globally important information through residual connections, and extracts features along the respective H and W directions through average pooling and concatenation operations to obtain the position information of relevant features, considering the feature locations. Finally, the introduction of the ODConv network enables it to pay more attention to the edge and blocked feature information in the image.

### 2.3. DeepSort Algorithm and Its Improvement

**Figure 7:**
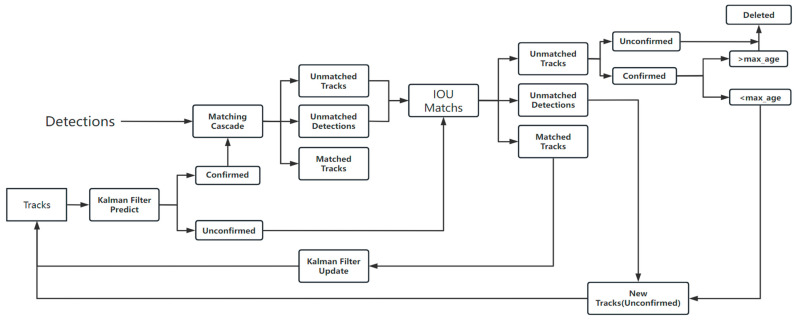
Algorithm flowchart of DeepSort model [28].

The DeepSort algorithm is an improved version of the Sort target tracking algorithm. The latter mainly includes the Hungarian matching algorithm and Kalman filter, which can combine the tracking and actual detection results to obtain the IOU (Intersection over Union) [35] and calculate the cost matrix to further detect and track the targets in the video. The former adds a discriminative network to the Sort algorithm and complements it with two steps of cascade matching and trajectory predictions, as shown in Figure 7.

#### 2.3.1. Cascade Matching

**Figure 8:**
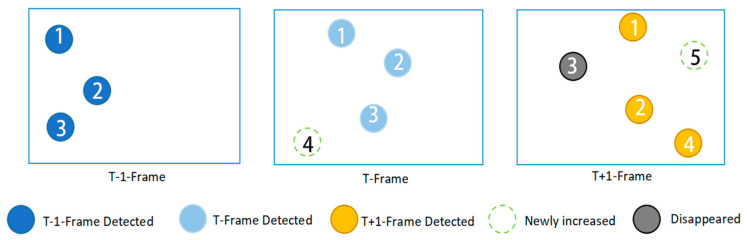
Algorithm flowchart of the DeepSort model’s matching process.

The DeepSort model’s tracking process is presented in Figure 8. The detection results obtained for the YOLOv7 model in this study were delivered to the tracking module to construct a link between the frames. For example, if the wheat appeared in frame T-1, the algorithm gave it an ID value of 1. Thereafter, the wheat ID remained as 1 for its subsequent appearances in other frames.

To track the wheat ears identified by the detection model, the DeepSort model uses an 8-dimensional variable *x* to describe the appearance and location of the detected wheat, as shown in Equation (4):

(4) $$x=(\mu ,\nu ,\gamma ,h,\dot{\mu },\dot{\nu },\dot{\gamma },\dot{h})$$

where (μ,ν) represents the center of the wheat, γ represents the aspect ratio of the wheat-ear-detection frame, h represents the height of the detection frame, and $\left(\dot{\mu },\dot{\nu },\dot{\gamma },\dot{h}\right)$ represents the corresponding speed of the wheat-detection frame’s movement in the video.

The DeepSort algorithm combines the wheat motion information with its appearance and then matches the prediction and detection frames using the Hungarian algorithm. To obtain the motion information, the martingale distance is used to describe the correlation between the Kalman filter prediction and the YOLOv7 detection results, as shown in Equation (5):

(5) $$d_{\left(i,j\right)}^{\left(1\right)}={\left(d_{j}-Y_{i}\right)}^{T}S_{i}^{-1}(d_{j}-Y_{i})$$

where *d_j_* refers to the jth YOLOv7 detection frame, *Y_i_* represents the state vector of the ith detection frame, and *S_i_* represents the standard deviation matrix between the i motion paths. Then, the Mahalanobis distance is used to screen the target. The Mahalanobis distance is less than the threshold *t*^(1)^ for a certain correlation, indicating the success of the motion state matching; otherwise, it fails, as shown in Equation (6):

(6) $$b_{\left(i,j\right)}^{\left(1\right)}=l[d^{\left(1\right)}(i,j)\leq t^{\left(1\right)}]$$

where *t*^(1)^ represents a relevant threshold, l = 1, $b_{\left(i,j\right)}^{\left(1\right)}$ represents a threshold indicator.

#### 2.3.2. Track Prediction

When the camera moves considerably, the Marcian distance is not a good measure of the degree of association, and ID jumps can lead to incorrect counting. To avoid such problems, the appearance feature information was used as the association information. A 128-dimensional feature vector *r_j_* is obtained for each detection target *d_j_*, and ‖*r_j_*‖ = 1 is specified as the constraint, while a feature vector that can predict its path after 100 frames is constructed for each wheat-ear, and then the minimum cosine distance between the feature descriptions of detection and tracking is calculated by Equation (7). the subsequent step compares the cosine distance with the correlation threshold obtained from training *t*^(2)^, similar to Equation (8), and if the result is less than the threshold value, it means that the association is successful.

(7) $$d_{\left(i,j\right)}^{\left(2\right)}=\min\{1-r_{j}^{T}r_{k}^{\left(1\right)}|r_{k}^{\left(1\right)}\in R_{i}\}$$

(8) $$b_{\left(i,j\right)}^{\left(2\right)}=l[d_{\left(i,j\right)}^{\left(2\right)}\leq t^{\left(2\right)}]$$

where *d*_(*i*,*j*)_ represents the minimum cosine distance, *r_j_* represents the feature vector of the detection frame, *r_k_* represents the feature vector successfully associated in the subsequent 100 frames, *R_i_* represents the set of appearance features, and *b*_(*i*,*j*)_ is the appearance indicator queue.

The above-mentioned minimum cosine distance can cause the lost target to reappear and recover its ID value, while the Marxian distance can provide a more reliable position prediction value in a short period of time. In order for the advantages of the two methods to complement each other, this paper combined Equations (6) and (8), which were linearly weighted, that is, we can obtain the threshold function to determine the success of the association, as shown in Equation (9):

(9) $$C_{\left(i,j\right)}=\lambda \cdot d_{\left(i,j\right)}^{\left(1\right)}+(1-\lambda )d_{\left(i,j\right)}^{\left(2\right)}$$

where λ is the weight value factor. Only when *C*_(*i*,*j*)_ is within the intersection of two *t*^(1)^ and *t*^(2)^ consecutive queues is the result considered as the correct match.

### 2.4. DeepSort Improvement

The original DeepSort appearance feature-extraction network uses a simple convolutional neural network consisting of only convolutional layers and residual components, which extracts the very limited deep appearance features of the target and cannot meet the task requirements of target appearance feature extraction in complex environments; therefore, this paper used ResNet to build a network model as the DeepSort appearance feature-extraction network, which increases the depth of the convolutional layer and helps to strengthen the ReID feature-recognition ability, creating a model with better performance and greater counting accuracy feature. The network structure is presented in Table 2.

The experiments used a pre-trained weight file on the Market-1501 dataset, which is suitable for the re-recognition of pedestrian appearance features, but not suitable for wheat-ear-recognition purposes. In order to improve the re-recognition effect for wheat ears, the re-recognition model was retrained using the relevant dataset by using the improved ResNet network structure to classify the wheat according to its external features, such as color, size, and texture.

## 3. Results

The experiment studies the accuracy of the proposed algorithm for video-based wheat-ear counting methods. It was divided into two stages: enhancing the detection effect of the small target through the improved YOLOv7 model for wheat ears that were relatively dense and heavily obfuscated, and tracking the effect of the same wheat ears in different video frames through the improved DeepSort algorithm.

### 3.1. Experimental Equipment and Parameter Settings

The experiments were conducted under a Linux Ubuntu 20.04.3 LTS operating system environment, with a 20G system disk and 50 GB NVME data disk for instant storage; NVIDIA GeForce RTX 3090 Ti GPU with a 24G memory size; and Pytorch 1.8.0 and CUDA 11.1 as deep learning frameworks. Detailed configuration information is presented in Table 3.

The parameter settings for the improved YOLOv7 model in the target-detection phase improved the model for identifying wheat ears using the Resnet model, and the DeepSort model used in the target tracking stage is presented in Table 4.

### 3.2. Evaluation Indicators

#### 3.2.1. Detection Algorithm Evaluation Index

The detection algorithm evaluation indexes used in this paper were: precision (*P*), recall (*R*), average precision (*AP*), and mean average precision (*mAP*), as shown in Equations (10)–(13):

(10) $$P=\frac{TP}{TP+FP}\times 100\%$$

(11) $$R=\frac{TP}{TP+FN}\times 100\%$$

(12) $$AP=\int_{0}^{1}P(R)dR$$

(13) $$mAP=\frac{\sum P_{A}}{N_{C}}$$

In the equations presented above, *TP* (true positive) represents the correct detection of a positive result; *FP* (false positive) represents a false-positive result; *FN* (false negative) represents a false-negative result, and *AP* (average precision) represents the average precision for each factor. *mAP* is the average of the *AP* values for each result. The closer the value is to 1, the better the detection capability. *Nc* represents the detection target type. In this case, the *Nc* value is 2, as the task of calculating the number of wheat ears requires the identification of wheat and non-wheat ears.

#### 3.2.2. Tracking Algorithm Evaluation Metrics

The evaluation indexes of the tracking algorithm used in this paper were: *M_m_* (miss detection rate), *M_f_* (false detection rate), *IDS* (ID switch), *MOTA* (multiple-object tracking accuracy), as shown in Equations (14)–(16):

(14) $$M_{m}=\frac{FN}{FN+TP}\times 100\%$$

(15) $$M_{f}=\frac{FP}{FP+TN}\times 100\%$$

(16) $$MOTA=1-\frac{\sum_{t}M_{m}+M_{f}+IDS}{\sum_{t}GT_{t}}$$

In the equations presented above, *IDS* represents the number of code conversions, *GT_t_* represents the number of targets, *d*_(*t*,*i*)_ represents the average metric distance between the target *i* and labeled box, and *c_t_* represents the number of the frame *t* matching results.

### 3.3. Target-Detection Results and Analysis

**Figure 9:**
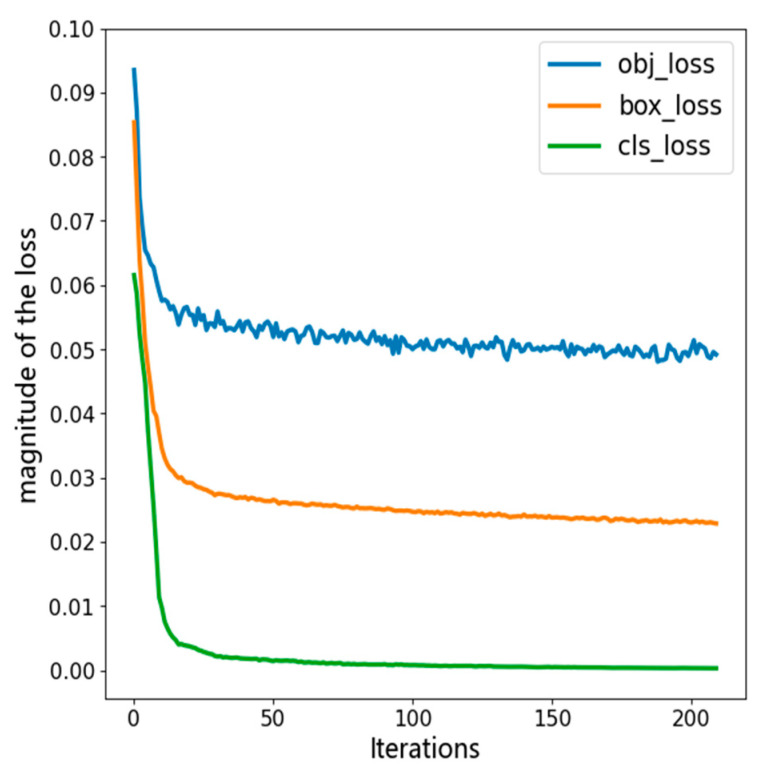
Loss function curve of improved YOLOv7 model.

The input image size of the training, test, and validation sets was 640 × 640, and training was performed in the built of improved YOLOv7 model. The convergence of the improved YOLOv7 model is shown in Figure 9. It can be concluded that the category, confidence, and position losses of the improved model converged to lower values, and the position loss decreased by 0.01, compared to the original model. The improved model’s detection algorithm converged better than the original model, presenting a more striking generalization performance.

A contrast experiment related to the improved algorithm and the original YOLOv7 model was performed to verify the efficiency of the improved algorithm for wheat-detection purposes and the rationality behind selecting the global feature network GCNet.

**Figure 10:**
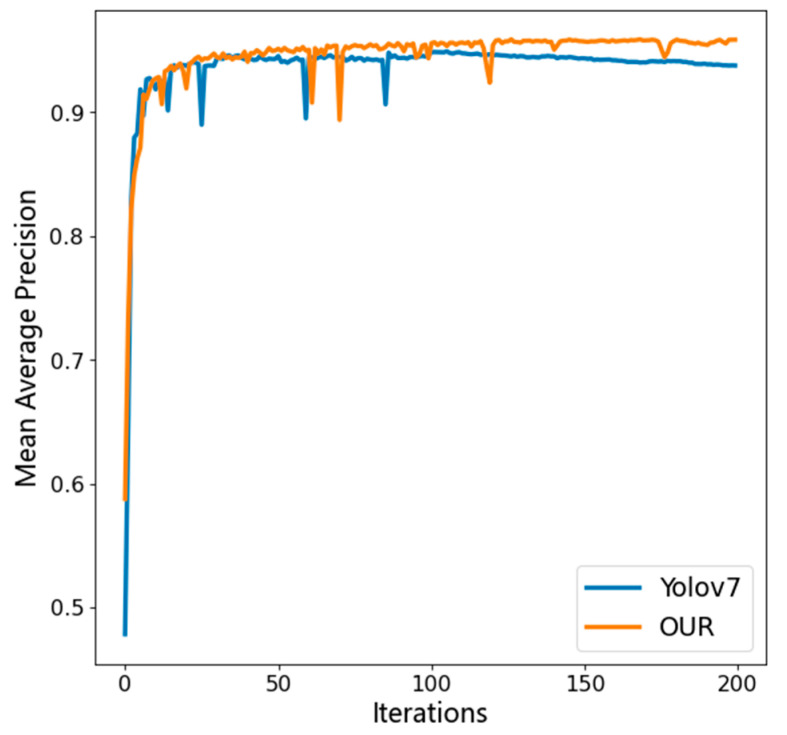
Accuracy rate curve of imporved YOLOv7 model.

As can be observed in Figure 10, the original YOLOv7 algorithm increases to 93.9% in 100 rounds of mAP and eventually converges at approximately 93.7%; while the algorithm proposed in this paper increased to 95.8% in 100 rounds of mAP and eventually converges at approximately 96.2%, which is a 2.5% improvement over the original YOLOv7 model. In brief, the accuracy of wheat-ear-detection methods can be increased without the participation of redundant parameters when the GCNet network’s architecture for collecting global information is incorporated into the network. Moreover, the introduction of ODConv and CA attention mechanisms enabled the model to consider dynamics in multiple dimensions and capture information across different latitudes, which is more conducive to the localization and identification of wheat sheaves and optimizes the target-detection performance.

To test the validity of each improvement point presented in this paper, ablation experiments were conducted using the YOLOv7 model as a reference in an environment where the parameters of each model were consistent. The results are presented in Table 5.

From Table 5, it can be observed that the YOLOv7 model increases the mAP value by 2.0% and the accuracy by 1.4% after the introduction of the lightweight network GCNet, while the number of parameters only increases by 1.3%; in model 3, after adding the ODConv method to the feature-extraction step, both the accuracy and mAP values increase by 0.2%; after adding the CA mechanism to the torso network in model 4, the accuracy and mAP values decrease by 0.5% and the model’s performance also decreases; and, after adding both ODConv and CA to the torso network, mAP increases to 96.2% and the accuracy increases to 93.5%. In summary, after adding GCNet to YOLOv7, the detection performance significantly improved; when only adding ODConv or CA mechanisms, the mAP value did not change considerably after the convergence stage and the network’s performance was limited. After adding both ODConv and CA mechanisms to the network, the number of parameters in the model slightly increased; however, compared with the YOLOv7 model, the network-detection performance significantly improved, laying a better foundation for the implementation of wheat-tracking and -counting behaviors.

**Figure 11:**
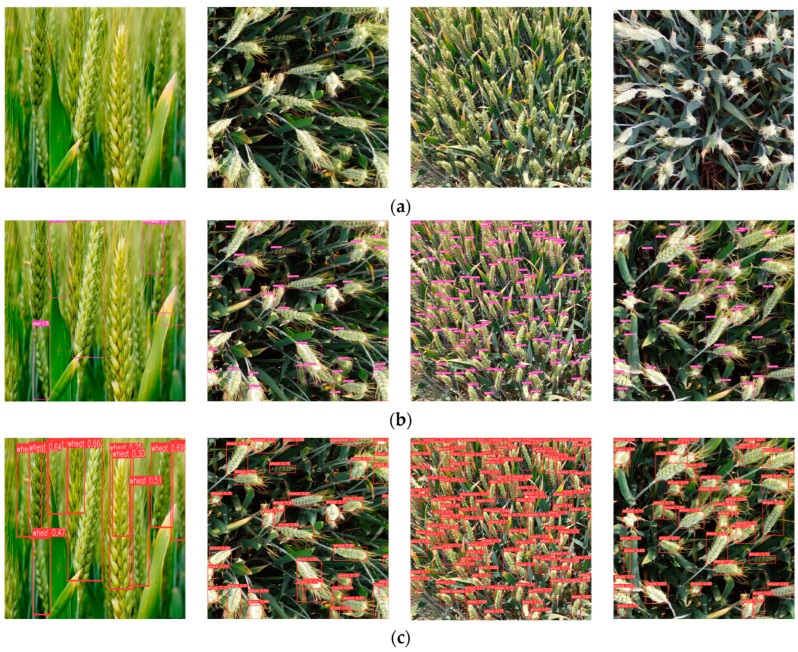

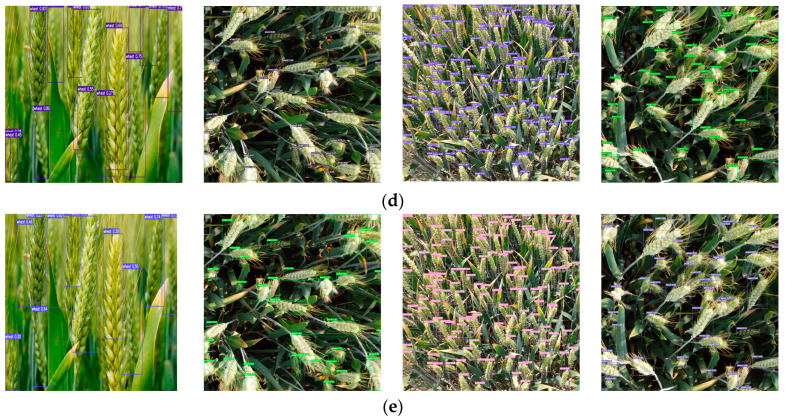
Comparison of detection effects of different algorithms. (**a**) Original images: numbers of wheat ears are 11, 40, 140, and 45, respectively. (**b**) Faster-RCNN: numbers of wheat ears are 6, 38, 133, and 44, respectively. (**c**) YOLOv5: numbers of wheat ears are 11, 37, 136, and 42, respectively. (**d**) YOLOv7: numbers of wheat ears are 13, 39, 134, and 43, respectively. (**e**) Our algorithms: numbers of wheat ears are 13, 39, 134, and 43, respectively.

To address the detection effect of the algorithm, this paper employed Faster-RCNN, YOLOv5s, YOLOv7, and the above-mentioned algorithm to conduct verifications in four situations, including severe wheat-ear shading, different wheat sizes, dense distribution, and uneven light. The results are shown in Figure 11.

From Figure 11, it can be concluded that the confidence level of the improved algorithm in detecting wheat ears was improved, in general. In the first group of experiments, it can be observed that the situation of a mutual overlay was serious, and the phenomena of the missing and false detections of individual wheat ears appeared in Faster-RCNN, YOLOv5, and YOLOv7 models. In this algorithm, the global information was encoded by adding GCNet to enhance the feature-extraction step, and the occluded wheat ears were well-distinguished and -recognized. In the second and third groups of experiments, the comparison determined that, except for the algorithm presented in this paper, the results were all affected by the variety and dense distribution of wheat, and it was impossible to accurately detect the distribution of the wheat ears. However, the addition of the CA mechanism and ODConv to this algorithm improved the location information of the wheat ears, enhanced the dynamics between each dimension, promoted the ability of the model to extract deep and shallow features globally, and effectively improved the ability to detect wheat ears. In the fourth group of experiments, it can be observed that the improved algorithm can achieve better results for light and dark changes, as well. To sum up, the algorithm proposed can detect wheat ears in large fields more accurately and can be applied to various distributions of wheat and complex field environments, further improving the detection accuracy compared to mainstream detection algorithms used in the research.

### 3.4. Results and Analysis of Video-Based Wheat-Crop Counting

#### 3.4.1. Re-Identification Experiments and Analysis of Results

The wheat re-recognition model created by training extracted and preserved the distinguishing features of wheat ears, and re-identified the same wheat ears appearing in different frames. Loss and Top-1 accuracy values were used to perform the evaluation, where Top-1 accuracy indicates the ratio of the total number of correct prediction probabilities of the model to all samples, and a value closer to 1 indicates that the model is more capable of extracting features, i.e., the better the re-identification of wheat ears.

**Figure 12:**
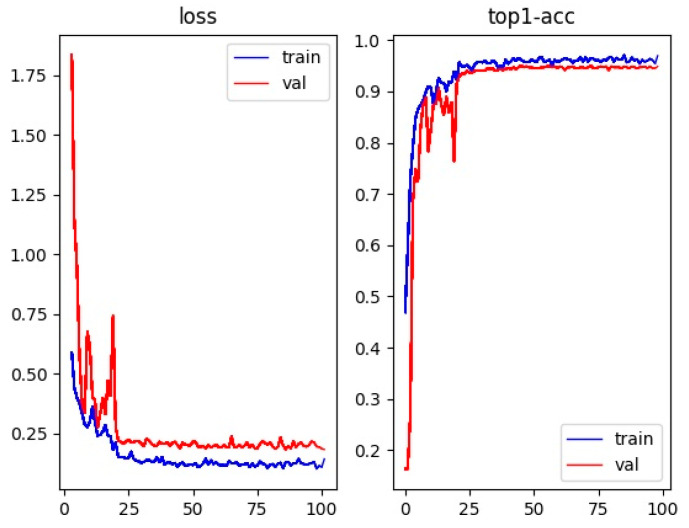
Convergence curves of detection loss values and Top-1 accuracy curves of wheat heads.

Figure 12 shows the wheat-ear convergence curve of the detection loss value and Top-1 accuracy curve of wheat ears. After 30 iterations of the model, both curves tended to be smooth, and the model basically achieved convergence; after 100 iterations of the model, the difference between the loss and accuracy values of the training and test sets was minor, and there was no evidence of the overfitting phenomenon; the loss value was 0.169 for the test set and the Top-1 accuracy rate reached 95.59%. At this time, the re-identification model obtained a weight file suitable for extracting the appearance features of the wheat ears, which can accurately re-identify the wheat ears.

#### 3.4.2. Wheat Spike Tracking Results and Count Analysis

In this paper, a detection algorithm with higher accuracy results was proposed to lay the foundation for the tracking and counting of wheat ears. In order to verify the performance of the improved algorithm in the tracking and counting of wheat ears, it was validated on a self-constructed dataset of wheat ears present in a large field. Ablation experiments were conducted to compare and verify the two stages of improvement with model performance changes. The results are shown in Table 6.

From Table 6, it can be concluded that the improved YOLOv7 model effectively improves the detection accuracy for wheat ears, while the detection speed does not change significantly. The improvement of the DeepSort model reduces the ID jump and enhances the performance of the tracker in detecting wheat ears and maintaining the wheat movement trajectory. The improved detection and tracking algorithms used in this study achieved 86.3% accuracy, 89.7% recall, 75.4% tracking accuracy, and 14 FPS tracking rate, which can better meet the requirements of tracking and counting wheat ears.

**Figure 13:**
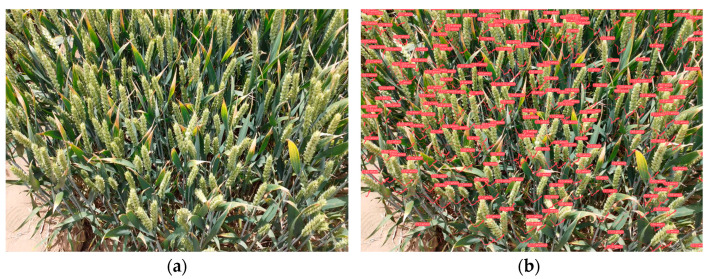
(**a**) Low-altitude video of wheat ears with (**b**) detection effects.

This study conducted verifications using videos shot by UAVs at a low altitude, to demonstrate the detection and tracking algorithms of wheat ears in a large field. The number of wheat was counted according to different ID numbers appearing in the video. It can be observed in Figure 13 that the left half is the actual field environment and the right half is the tracking count of the algorithm for wheat. A unique ID was provided to every wheat sample for tracking, and the number of wheat ears in the field could be obtained by counting the number of IDs assigned to them by the unmanned aerial vehicle during its flight.

As shown in Figure 13, although most of the wheat ears were correctly detected and tracked by the model presented in this paper, some of them remain untracked or detected in a correct manner. This was mainly due to two reasons: on the one hand, some of the wheat ears in the video were severely obstructed or poorly lit; on the other hand, the video sometimes presented a prolonged motion with a variable speed so that the DeepSort model was unable to track the identified wheat ears correctly.

**Figure 14:**
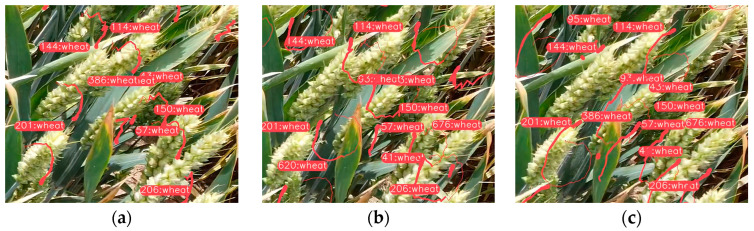
Tracking effect on wheat in the video: (**a**) 50th, (**b**) 75th, and (**c**) 100th frames.

As shown in Figure 14, the captured video constantly shook during the movement of the UAV, and the wheat ears detected in the previous frames retained their unchanged IDs. For the newly detected wheat ears, the ID changed due to the constant accumulation of tracking values; however, the algorithm we proposed recorded the number of different IDs and did not affect the automatic counting of the wheat.

According to the growth cycle, distribution, and environmental aspects of the wheat ears, the following five validation videos were selected from the tracking and counting videos. The characteristics of videos 1~5 were wheat field at maturity, wheat field at filling stage, obvious shading phenomenon among wheat, presence of light and dark in the environment, and dense distribution of wheat. The L1 loss and accuracy were selected as the metric values to count the wheat ears appearing in the video, and the results are shown in Table 7.

From Table 7, it can be observed that the average value of the L1 loss obtained from counting wheat ears in five videos with different characteristics is 4.2, and the accuracy is between 95 and 98%. The algorithm presented in this paper has an outstanding tracking and counting result for wheat ears, which can play a central role in guiding wheat yield predictions.

## 4. Conclusions

The direct counting of wheat ears in a field was achieved through videos shot by a UAV using improved YOLOv7 DeepSort models, which are more efficient and suitable for counting wheat ears in large fields, compared to the static-image-based counting method.

In wheat-ear-detection methods, images of wheat ears during different growth periods and complex natural environments are used as datasets to improve the robustness of wheat-ear-detection methods. The combination of GCNet, ODConv, and CA mechanisms in the YOLOv7 model produces better feature extraction for wheat ears and improves detection accuracy. In terms of target tracking, based on the DeepSort algorithm, the feature-extraction network was improved and ResNet was used to replace the original CNN network structure so that the DeepSort algorithm could strengthen the recognition ability of the model for wheat in the tracking process and produce a better tracking result.

The method proposed in this paper was verified based on the wheat-ear dataset collected by the UAV. The results show that the accuracy, recall, and mAP results of the method proposed for wheat-detection purposes in the test set were 93.5%, 92.4%, and 96.2%, respectively. The accuracy of the multi-objective tracking algorithm was 86.3%, which was 17.1% higher than before the improvement, and the detection rate reached 14 frames per second, presenting a good real-time performance, and MOTA was 75.4%, 30.1% higher than before the improvement. In the extracted video displaying wheat-ear counting, the counting accuracy was stable above 95%.

The algorithm proposed in this paper was applicable to wheat field videos at nearly uniform speeds; however, the counting accuracy can be reduced in videos with variable speeds, together with a high requirement for good video definition. In the following studies, we will continue to explore wheat-detection and -tracking methods in scenarios where wheat ears are severely obstructed or appear in highly dense formations, and we will adopt a lightweight model to achieve the same or better detection accuracies, reduce the quality requirements for the video dataset, and reduce the impact of sharp inter-frame motion on the counting accuracy. The proposed algorithm can be deployed on UAVs or other edge devices for the task of rapid wheat counting.

## Figures and Tables

**Table 1:** Composition of the dataset.

Data Sources	Data Contents	Dataset Construction
GWHD	4700 high-resolution RGB images	Select 2600 images with significant differences
	190,000 labeled wheat ears	110,000 labeled wheat ears
Self-built datasets	117 min video of wheat ears	Screen out 8 min constant-speed videos
	20,000-frame wheat-ear images	Screen out 4200-frame images

**Table 2:** Network structure of the improved ResNet model.

Name	Patch Size/Stride	Output Size
Conv1	3 × 3/1	32 × 128 × 64
Conv2	3 × 3/1	32 × 128 × 64
Max Pool 3	3 × 3/2	32 × 64 × 32
Residual 4	3 × 3/1	32 × 64 × 32
Residual 5	3 × 3/1	32 × 64 × 32
Residual 6	3 × 3/1	32 × 64 × 32
Residual 7	3 × 3/1	32 × 64 × 32
Residual 8	3 × 3/2	64 × 32 × 16
Residual 9	3 × 3/1	64 × 32 × 16
Residual 10	3 × 3/1	64 × 32 × 16
Residual 11	3 × 3/1	64 × 32 × 16
Residual 12	3 × 3/1	64 × 32 × 16
Residual 13	3 × 3/1	64 × 32 × 16
Residual 14	3 × 3/2	128 × 16 × 8
Residual 15	3 × 3/1	128 × 16 × 8
Residual 16	3 × 3/1	128 × 16 × 8
Residual 17	3 × 3/1	128 × 16 × 8
Residual 18	3 × 3/2	256 × 8 × 4
Residual 19	3 × 3/1	256 × 8 × 4
Residual 20	3 × 3/1	512 × 8 × 4
Residual 21	3 × 3/1	512 × 8 × 4
AdaptiveAvgPool2d	512	
Batch and normalization	512	

**Table 3:** Detailed configuration information of the experimental environment.

Configuration	Parameters
Operating system	Linux Ubuntu 20.04.3 LTS
Capacity of memory	System Disk 20G, Data Disk 50 GB NVME
GPU	A5000-24G
CPU	AMD
Model framework	Pytorch 1.8.0 and CUDA 11.1
Programming languages	Python3.8

**Table 4:** Parameter settings of each model stage.

Various Stages of the Model	Configuration	Parameters
Wheat-ear detection	Optimizer	Stochastic gradient descent
	Batch_size	24
	Initial Learning Rate	0.01
	Momentum	0.937
	Weight_decay	0.0005
Training of wheat-ear re-identification	Input Image Size	128 × 64
	Batch_size	64
	Epochs	300
Wheat-ear tracking	MAX_DIST	0.3
	NMS_MAX_OVERLAP	0.5
	MIN_CONFIDENCE	0.2
	MAX_IOU_DISTANCE	0.8
	N_INIT	5

**Table 5:** Results of ablation experiments with different modules.

Models	GCNet	ODConv	CA	Precision	Recall	mAP	Parameters (M)
1				91.9%	92.1%	93.7%	37.20
2	√			93.3%	92.3%	95.7%	37.56
3	√	√		93.1%	92.7%	95.9%	39.70
4	√		√	92.8%	91.3%	95.2%	37.33
5	√	√	√	93.5%	92.4%	96.2%	40.48

**Table 6:** Results of ablation experiments with different combinations.

Framework	Precision/%	Recall/%	MOTA/%	FPS (f·s^−1^)
YOLOv7-DeepSort	69.2	65.8	45.3	16
Improved YOLOv7-DeepSort	80.7	85.7	68.4	14
YOLOv7-Improved DeepSort	74.9	71.2	51.2	15
Algorithm of this paper	86.3	89.7	75.4	14

**Table 7:** Results of counting wheat ears under different conditions.

	Video	01	02	03	04	05
Indicators						
Projections/true		44/46	82/87	39/41	77/81	310/318
L1 loss		2	5	2	4	8
Accuracy		95.7	96.6	95.1	95.1	97.5

## Data Availability

The raw data supporting the conclusions of this article will be made available by the authors, without undue reservations.
